# Agonist-Triggered Ca^2+^ Release From Functionally Connected Endoplasmic Reticulum and Lysosomal Ca^2+^ Stores in bEND.3 Endothelial Cells

**DOI:** 10.33549/physiolres.935472

**Published:** 2025-04-01

**Authors:** Cing-Yu CHEN, Yu-Jen CHEN, Cheng-An WANG, Chen-Hsiu LIN, Jong-Shiuan YEH, Paul CHAN, Lian-Ru SHIAO, Yuk-Man LEUNG

**Affiliations:** 1 Department of Cosmetic Science, Providence University, Taichung, Taiwan; 2 School of Pharmacy, China Medical University, Taichung, Taiwan; 3 Division of Cardiovascular Medicine, Department of Internal Medicine, Wan Fang Hospital, Taipei Medical University, Taipei, Taiwan; 4 Division of Cardiology, Department of Internal Medicine, School of Medicine, College of Medicine, Taipei Medical University, Taipei, Taiwan; 5 Taipei Heart Institute, Taipei Medical University, Taipei, Taiwan; 6 Institute of Public Health, National Yang Ming Chiao Tung University, Taipei, Taiwan; 7 Graduate Institute of Clinical Medicine, College of Medicine, Taipei Medical University, Taipei, Taiwan; 8 Department of Physiology, China Medical University, Taichung, Taiwan

**Keywords:** Endothelial cells, Lysosome, Endoplasmic reticulum, Ca^2+^ release

## Abstract

Endoplasmic reticulum (ER) and lysosomes are physiologically active, physically and functionally connected intracellular Ca^2+^ stores. In this study we investigated agonist-triggered Ca^2+^ release from these two stores in mouse microvascular endothelial bEND.3 cells. Addition of nigericin to discharge lysosomal Ca^2+^ did not affect endoplasmic reticulum Ca^2+^ release induced by cyclopiazonic acid (CPA) and vice versa, suggesting lysosomes and ER were separate Ca^2+^ stores whose Ca^2+^ content was not readily reduced by depletion of the counterpart. ATP-triggered Ca^2+^ release was partially inhibited by Ned-19 (lysosomal two-pore channel inhibitor) or xestospongin C (inositol 1,4,5-trisphosphate receptor-channel inhibitor), suggesting ATP mobilized Ca^2+^ from both ER and lysosomes. Whilst ATP-triggered Ca^2+^ release did not affect subsequent CPA- or nigericin-induced Ca^2+^ discharge, pretreatment with either CPA or nigericin abolished subsequent ATP-triggered Ca^2+^ release. Thus, the empty state of ER suppressed lysosomal Ca^2+^ release elicited by ATP, and vice versa, the empty state of lysosome inhibited ATP-triggered Ca^2+^ release from ER. These data suggest cross-talk of the two organelles on the Ca^2+^ filling state to regulate agonist-stimulated Ca^2+^ release of each other.

## Introduction

Many hormones, through activating G-protein (G_q/11_)-coupled receptors that are linked with phospholipase C, generates inositol-1,4,5-trisphosphate (IP_3_) and diacylglycerol (DG) from phosphatidyl-4,5-bisphosphate (PIP_2_) cleavage. IP_3_ mobilizes Ca^2+^ from intracellular Ca^2+^ store (endoplasmic reticulum; ER), while DG activates protein kinase C [1]. After Ca^2+^ mobilization from the store by IP_3_, the emptiness of the Ca^2+^ store would trigger the opening of a store-operated Ca^2+^ channel (SOCC) at the plasma membrane; this SOCC is composed of the proteins STIM (as a sensor of Ca^2+^ content in the store) and Orai (the channel protein molecule at the plasma membrane) [2]. More recent evidence suggests SOCC may also involve TRPCchannels [3].

In addition to the endoplasmic/sarcoplasmic reticulum as a major intracellular Ca^2+^ reservoir [4], lysosomes are also physiologically active Ca^2+^ stores [5–7]. Expressed on the membrane of the lysosomes are nicotinic acid adenine dinucleotide phosphate (NAADP)-gated two-pore channels (TPC); when the latter open, Ca^2+^ is released into the cytosol [8]. NAADP has been well demonstrated to be a potent Ca^2+^-mobilizing second messenger in a wide range of cell types, in addition to the other two well-known ones, IP_3_ and cADP ribose [9]. Pharmacologically, the acidic lysosomal Ca^2+^ stores could be discharged by nigericin, which dissipates the H^+^ gradient across lysosomal membranes and thus prevents Ca^2+^ from refilling the stores.

Cellular Ca^2+^ responses often involve agonist-triggered Ca^2+^ release from both ER and lysosomes. For instance, Zuccolo *et al*. [5] have shown that in brain endothelial cells, glutamate-triggered Ca^2+^ oscillations result from Ca^2+^ release elicited by both IP_3_ and NAADP. In this study, we showed that ATP stimulated Ca^2+^ release from both ER and lysosomes in mouse bEND.3 endothelial cells; we also demonstrated the functional connection between these two Ca^2+^ release processes.

## Materials and Methods

### Cell culture and materials

Dulbecco’s modified Eagle’s medium (DMEM), fetal calf serum, and tissue culture reagents were purchased from Invitrogen Corporation (Carlsbad, CA, USA). ATP and cyclopiazonic acid were from Sigma-Aldrich (MA, U.S.A). Nigericin, Ned-19 and xestospongin C were purchased from Tocris Bioscience (Bristol, U.K.). DMSO (≥99.7 %) was used to dissolve CPA, Ned-19 and xestospongin C to yield stock solutions of 30 mM, 3 mM and 2 mM, respectively; ethanol (≥99.8 %) was used to dissolve nigericin to yield a stock solution of 30 mM. Fura-2 AM was purchased from Calbiochem-Millipore. bEND.3 cells were cultured in Dulbecco’s modified Eagle’s medium (DMEM) supplemented with 10 % fetal bovine serum and 1 % penicillin/streptomycin.

### Microfluorimetric measurement of cytosolic Ca^2+^

Microfluorimetric measurement of cytosolic Ca^2+^ concentration was performed using fura-2 as a Ca^2+^-sensitive fluorescent dye as previously reported [10]. In brief, cells were incubated with 5 μM fura-2 AM (Invitrogen) for 1 h at 37ºC and then washed in extracellular bath solution which contained (mM): 140 NaCl, 4 KCl, 1 MgCl_2_, 2 CaCl_2_, 10 HEPES (pH 7.4 adjusted with NaOH). When intracellular Ca^2+^ release was assayed, Ca^2+^-free solution was used. This Ca^2+^-free solution was the same as the extracellular bath solution mentioned above except that Ca^2+^ was omitted and 100 μM EGTA was supplemented. Cells were alternately excited with 340 nm and 380 nm (switching frequency at 1 Hz) using an optical filter changer (Lambda 10-2, Sutter Instruments). Emission was collected at 500 nm and images were captured using a CCD camera (CoolSnap HQ2, Photometrics, Tucson, AZ) linked to an inverted Nikon TE2000-U microscope. Images were analyzed with an MAG Biosystems Software (Sante Fe, MN). All imaging experiments were performed at room temperature (25 ºC). We measured and analyzed the 340/380 ratio changes at a region of interest of single cells within the microscopic views (regarded as one experiment) and then repeated this experiment a few more times to get the mean of all single cells examined.

### Statistical Analysis

Data are presented as means ± SEM. ANOVA was used to compare multiple groups, followed by the Tukey’s HSD post-hoc test. A value of p<0.05 is considered significantly different.

## Results

**Fig. 1:**
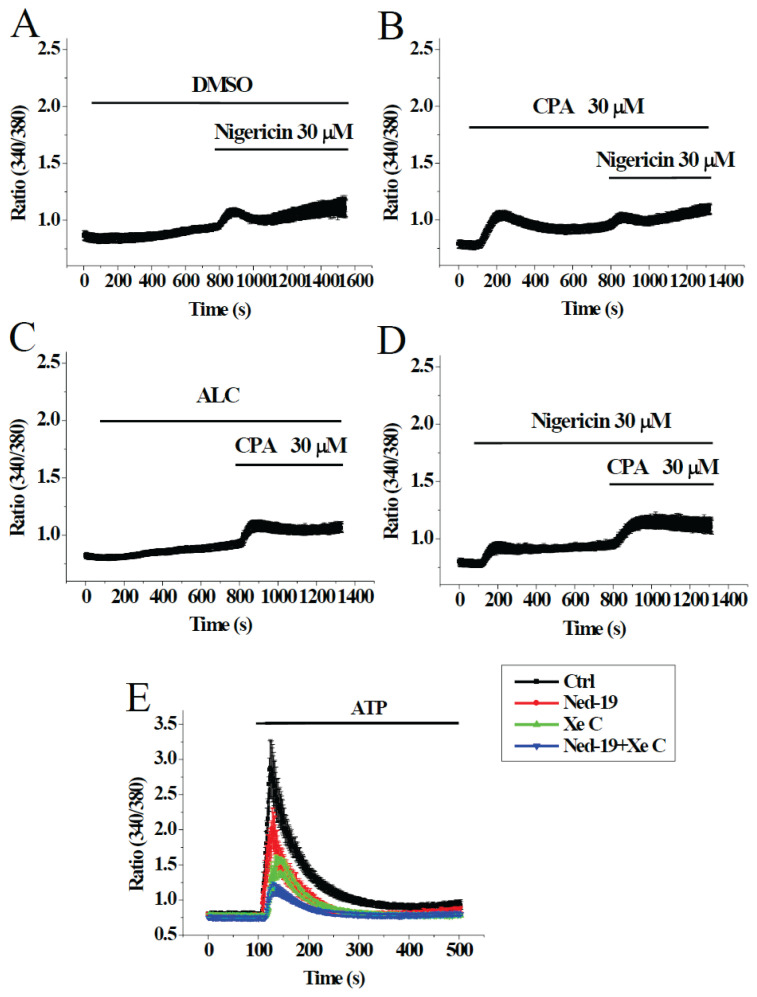
Agonist-triggered Ca^2+^ release from ER and lysosomes. [Ca^2+^] was measured in bEND.3 cells bathed in Ca^2+^-free solution. (**A,B**) Cells were stimulated by DMSO (vehicle control) or 30 μM CPA (which caused ER Ca^2+^ release) and then 30 μM nigericin (which caused lysosomal Ca^2+^ release). (**C,D**) Cells were stimulated by ethanol as vehicle control (alcohol, ALC) or 30 μM nigericin and then30 μM CPA. Results are mean ± SEM of 7–29 cells from 3–4 separate experiments. (E) Cells were pretreated with DMSO, 2 μM XeC (IP_3_ receptor-channel inhibitor), 3 μM Ned-19 (lysosome TPC inhibitor), or a combination of the two agents for 8 min before stimulated by 10 μM ATP. The peak Ca^2+^ response of the control group (DMSO) was significantly different (p<0.05) from the other three groups. Results are mean ± SEM of 22–45 cells from 3 separate experiments.

As shown in Fig. 1A, addition of nigericin caused Ca^2+^ release, indicating the existence of lysosomal Ca^2+^ stores. After addition of CPA to cause endoplasmic reticulum Ca^2+^ release, subsequent nigericin treatment still caused Ca^2+^ release (Fig. 1B). As shown in Fig. 1C and D, CPA-triggered Ca^2+^ release was not reduced by pretreatment with nigericin. Taken together, these data suggest the Ca^2+^ pools released by CPA (endoplasmic reticulum) and nigericin (lysosomes) were largely separate. To show whether agonist-triggered Ca^2+^ signaling involved lysosomal Ca^2+^ stores, we pretreated bEND.3 cells with 3 μM Ned-19 (lysosome TPC inhibitor), 2 μM xestospongin C (XeC, IP_3_ receptor-channel inhibitor), or a combination of Ned-19 and XeC, before ATP stimulation in Ca^2+^-free solution (Fig. 1E). Results show that pretreatments with Ned-19 or XeC could partially suppress ATP-triggered Ca^2+^ release; pretreatment with both agents caused additive suppression. These data suggest ATP mobilized Ca^2+^ from both ER and lysosomes.

**Fig. 2:**
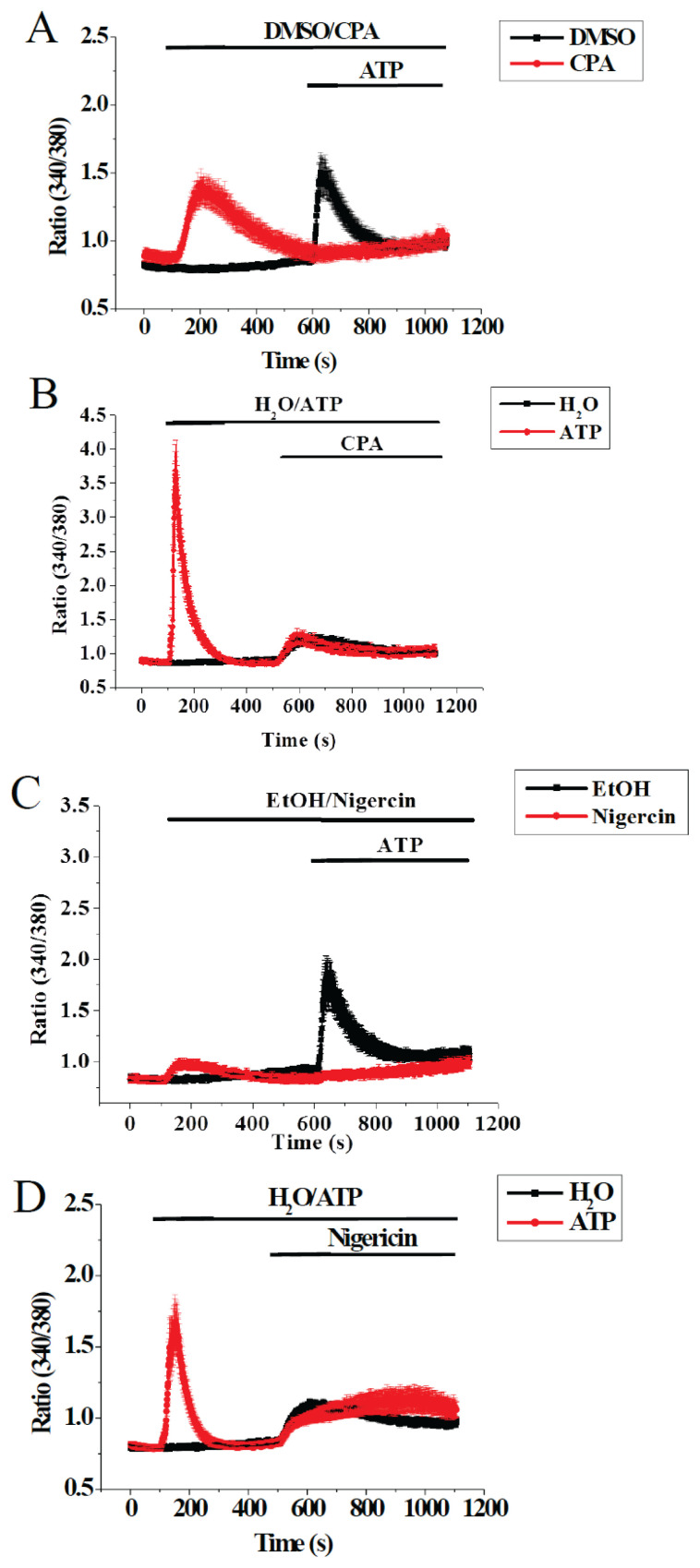
Emptying CPA- or nigericin-sensitive pools abolished ATP-triggered Ca^2+^ release. [Ca^2+^] was measured in bEND.3 cells bathed in Ca^2+^-free solution. (**A**) Cells were pretreated with DMSO or 30 μM CPA (which caused ER Ca^2+^ release) before stimulated by 10 μM ATP. (**B**) Cells were pretreated with water or 10 μM ATP before stimulated by 30 μM CPA. (**C**) Cells were pretreated with ethanol (EtOH) or 30 μM nigericin (which caused lysosomal Ca^2+^ release) before stimulated by 10 μM ATP. (**D**) Cells were pretreated with water or 10 μM ATP before stimulated by 30 μM nigericin. Results are mean ± SEM of 29–35 cells from 3–4 separate experiments.

We proceeded to demonstrate that ATP-triggered Ca^2+^ release did come from both ER and lysosomes. As shown in Fig. 2A, CPA pretreatment abolished subsequent ATP-triggered Ca^2+^ release. However, pre-exposure to ATP to release Ca^2+^ did not affect subsequent CPA-induced Ca^2+^ discharge (Fig. 2B), suggesting the ATP-sensitive pool resided in the much bigger CPA-sensitive pool. As shown in Fig. 2C, nigericin pretreatment abolished subsequent ATP-trig-gered Ca^2+^ release. However, pre-exposure to ATP to release Ca^2+^ did not affect subsequent nigericin-induced Ca^2+^ discharge (Fig. 2D), suggesting the ATP-sensitive pool resided in the much bigger nigericin-sensitive pool.

## Discussion

Results in our work suggest the ER and lysosomes were separate Ca^2+^ stores in bEND.3 cells: nigericin could mobilize lysosomal Ca^2+^ after CPA-induced Ca^2+^ discharge, and CPA could mobilize ER Ca^2+^ after nigericin-induced Ca^2+^ discharge (Fig. 1A–D). This is in contrast to the observation that pharmacologically depleting ER leads to diminished lysosomal Ca^2+^ content and vice versa in HeLa cells [11]. Data in Fig.1E indicate that ATP caused Ca^2+^ release from both the ER and lysosomes. It is therefore difficult to explain the absence of ATP response after CPA-induced Ca^2+^ release (Fig. 2A): ATP was expected to mobilize Ca^2+^ from lysosomes. Similarly, the absence of ATP response after nigericin-induced Ca^2+^ release was intriguing (Fig.2C): ATP was expected to mobilize Ca^2+^ from the ER. That ATP is able to mobilize ER Ca^2+^ after lysosomal Ca^2+^ discharge was observed in canine kidney MDCK cells: ATP-induced Ca^2+^ release was not abolished after lysosomal Ca^2+^ was released by glycyl-L-phenylalanine-beta-naphthylamide, an agent known to permeabilize lysosomes by osmotic swelling [12].

ER and lysosomes are physically and functionally connected [13–15]. For instance, Niemann Pick type C1 protein tethers ER and lysosomes to facilitate cholesterol transport [16]. Local NAADP-triggered Ca^2+^ release sensitizes neighboring ER IP_3_R to IP_3_-induced Ca^2+^ release, thus endowing local “Ca^2+^ blips” to propagate as global Ca^2+^ signaling [17]. Lysosomes provide a rapid sequestration of Ca^2+^ released via IP_3_R [6,18]. It is noted that TPC is not the only route of Ca^2+^ release from lysosomes. For instance, TRPML1 and TRPA1 have been shown to mediate lysosomal Ca^2+^ release in human fibroblasts and mouse dorsal root ganglion neurons, respectively [19,20]. The observation that pharmacologically depleting ER leads to diminished lysosomal Ca^2+^ content and vice versa in HeLa cells suggests Ca^2+^ flow (via TPC or IP_3_R) or reciprocal sensing (by yet unknown mechanisms) of Ca^2+^ balance across the ER-lysosome interface [11]. Our results, however, show that depletion of either store did not appear to significantly affect Ca^2+^ content of the other store in bEND.3 cells. Thus, ATP’s inability to release Ca^2+^ after either ER or lysosomal discharge was unlikely due to the emptiness of lysosomes (after CPA treatment) or ER (after nigericin treatment). Our results therefore suggest, via yet uncertain mechanisms, complete ER depletion suppressed NAADP-triggered Ca^2+^ release, and complete lysosomal depletion inhibited IP_3_-triggered Ca^2+^ release in bEND.3 cells. Ca^2+^ filling state of ER is transduced in a retrograde manner to the plasmalemma via STIM1-Orai1 interaction to regulate SOCC [2]. A link of ER/lysosomal emptiness to receptor-effector uncoupling as an alternative explanation for the absence of ATP response after ER or lysosomal Ca^2+^ discharge could not be ruled out. The above-mentioned putative store-store or store-plasmalemma linking mechanisms certainly warrant future verification.
